# Association Between Carotid Arterial Strain and Heart Rate Variability in Older Age

**DOI:** 10.1111/jch.70312

**Published:** 2026-06-15

**Authors:** Massimiliano Fornasiero, Matthew A. Stanley, Matt Webber, James C. Moon, Peter Friberg, Cristian Topriceanu, Alun D. Hughes, Gabriella Captur

**Affiliations:** ^1^ Institute of Cardiovascular Science University College London London UK; ^2^ UCL Medical School London UK; ^3^ Centre for Inherited Heart Muscle Conditions Cardiology Department The Royal Free Hospital, Hampstead London UK; ^4^ Cardiac MRI Unit Barts Heart Centre London UK; ^5^ Department of Physiology Institute of Medicine Sahlgrenska Academy, University of Gothenburg Gothenburg Sweden; ^6^ Unit For Lifelong Health and Ageing at UCL University College London London UK

**Keywords:** arterial stiffness, carotid intima‐media thickness, heart rate

## Abstract

Loss of the normal heart rate variability (HRV) predicts death. This study investigated the association between carotid arterial strain (CAS) and HRV in an older age population‐based cohort. We hypothesized that impaired arterial compliance and increased vascular stiffness could inhibit the baroreceptor reflex, resulting in cardiac autonomic dysfunction and loss of the normal HRV. Participants (60–64 years) were from the 1946 Medical Research Council, National Survey of Health and Development, British birth cohort. Carotid intima media thickness (cIMT) and CAS (exposures) were measured by ultrasound, and time‐ and frequency‐domain HRV indices (outcomes) by a resting 5‐min 12‐lead tachogram. Generalized linear models were used, adjusted for relevant clinic‐demographic confounders and subjected to sensitivity analysis in which we re‐analyzed associations after additional adjustment for cIMT and after removing participants with known cardiovascular disease. Eight hundred and ninety‐six participants were included. On univariate analysis, CAS was associated with HRV markers: standard deviation of normal‐to‐normal beats (SDNN), root mean square of successive differences (RMSDD), HRV triangular index, high‐ and low‐frequency (H/LF) normalized high‐frequency power (all *p *< 0.05). Associations persisted in fully confounder‐adjusted models: SDNN *β* = 0.52 [confidence interval: 0.2, 0.8] *p *< 0.001; RMSDD* β* = 0.59 [0.3, 0.9], *p *< 0.001; HRV triangular index *β* = −0.34 [−0.5, −0.1], *p *< 0.001; HF power *β* = 8.33 [2.2, 14.4], *p* = 0.007; LF power *b* = 8.47 [1.0, 15.9], *p* = 0.026; normalized HF power *β* = 0.55 [0.2, 0.9], *p* = 0.006. Key associations persisted in the sensitivity analysis. In conclusion, regardless of carotid atherosclerotic vascular disease (indicated by cIMT), hypertension, or stroke, our results demonstrated that carotid stiffening in older age associates with a loss of HRV, potentially through an impaired baroreceptor response.

## 1 Introduction

It has been suggested that reduced carotid arterial strain (CAS) caused by atherosclerosis, aging, and metabolic factors such as hyperglycaemia [1], reduces the sensitivity of carotid baroreceptors to blood pressure changes, resulting in cardiac autonomic dysfunction [2]. Such autonomic dysfunction is known to manifest, amongst other things, as a dampening of heart rate variability (HRV).

CAS is measured by ultrasound imaging and ultimately relates to the organization of collagen plus quantity and integrity of elastin in the arterial wall [3]. Alongside carotid intima‐media thickness (cIMT) measurement, CAS has been validated as an independent predictor of cardiovascular events [4]. It is considered one of the earliest markers of subclinical carotid artery atherosclerosis [5], demonstrating supplementary predictive value to cIMT measurement alone in broader cardiovascular risk prediction models [4, 6, 7].

Low HRV has been implicated in increased mortality, for example, following myocardial infarction [8] and in heart failure [9]. Indices of HRV are clinically obtained through time‐ and frequency‐domain analysis of electrocardiogram (ECG) traces, typically utilizing either short‐term (typically 5‐min resting ECG recordings) or long‐term recordings (typically via 24‐h Holter monitoring), as validated by the Foundational 1996 European Society of Cardiology Task Force Guidance on HRV analysis [10].

Short‐term HRV is considered to be driven by two overlapping processes: Firstly, a complex relationship between sympathetic and parasympathetic nerve branches; secondly, baroceptor (based in the carotid arteries and aortic arch) and vascular tone‐mediated regulatory mechanisms, driving changes in heart rate via respiratory sinus arrhythmia [11, 12]. Long‐term HRV is impacted by additional factors such as circadian rhythm, core body temperature, metabolism, sleep cycle, and the renin–angiotensin system [11]. While short‐term ECG HRV analysis has demonstrated utility in the prediction of outcomes, long‐term HRV analysis is considered the gold standard measurement with regard to cardiovascular prognostication [11, 13]. The benefit of using short‐term HRV analysis for research lies in the ability to better control for confounding factors under strict laboratory conditions, such as body position, physical activity, respiration, and environmental factors (that cannot be guaranteed with extended Holter monitoring) [12, 13]. Furthermore, short‐term HRV analysis using a 5‐min ECG has been revalidated as a highly reliable measure of autonomic function in young, active adults [12]. It also demonstrates proven efficacy in the assessment of HRV in older age throughout the wider literature [14, 15].

An association between carotid stiffening and short‐term HRV has been found in young patients with type 1 diabetes [16] and in young people free from cardiovascular disease [17], but whether this association persists into older age at the population level is not known. We sought to investigate whether CAS and cIMT were associated with HRV in an older age British‐based cohort.

## 2 Methods

### 2.1 Participants

Participants were from the Medical Research Council (MRC) National Survey of Health and Development (NSHD)—a birth cohort study comprised of 5362 individuals born in 1 week in 1946 in Britain. The cohort has been extensively followed up with periodic assessments, which have been described elsewhere [18]. Briefly, the cohort has been evaluated multidimensionally: anthropometrically, socio‐economically, and in terms of lifestyle choices (e.g., smoking) and health function (e.g., mental health, cardiovascular, and respiratory function) [18]. The current cross‐sectional study uses data collected between 2006 and 2010 when participants were aged 60–64 years of age. Written informed consent was obtained from all participants, and ethical approval was granted from the Greater Manchester Local Research Ethics Committee and the Scotland Research Ethics Committee [18]. All procedures were in accordance with the ethical standards of our institutional and/or national research ethics committees and conformed to the 1964 Helsinki declaration and its later amendments or comparable ethical standards.

**FIGURE 1:**
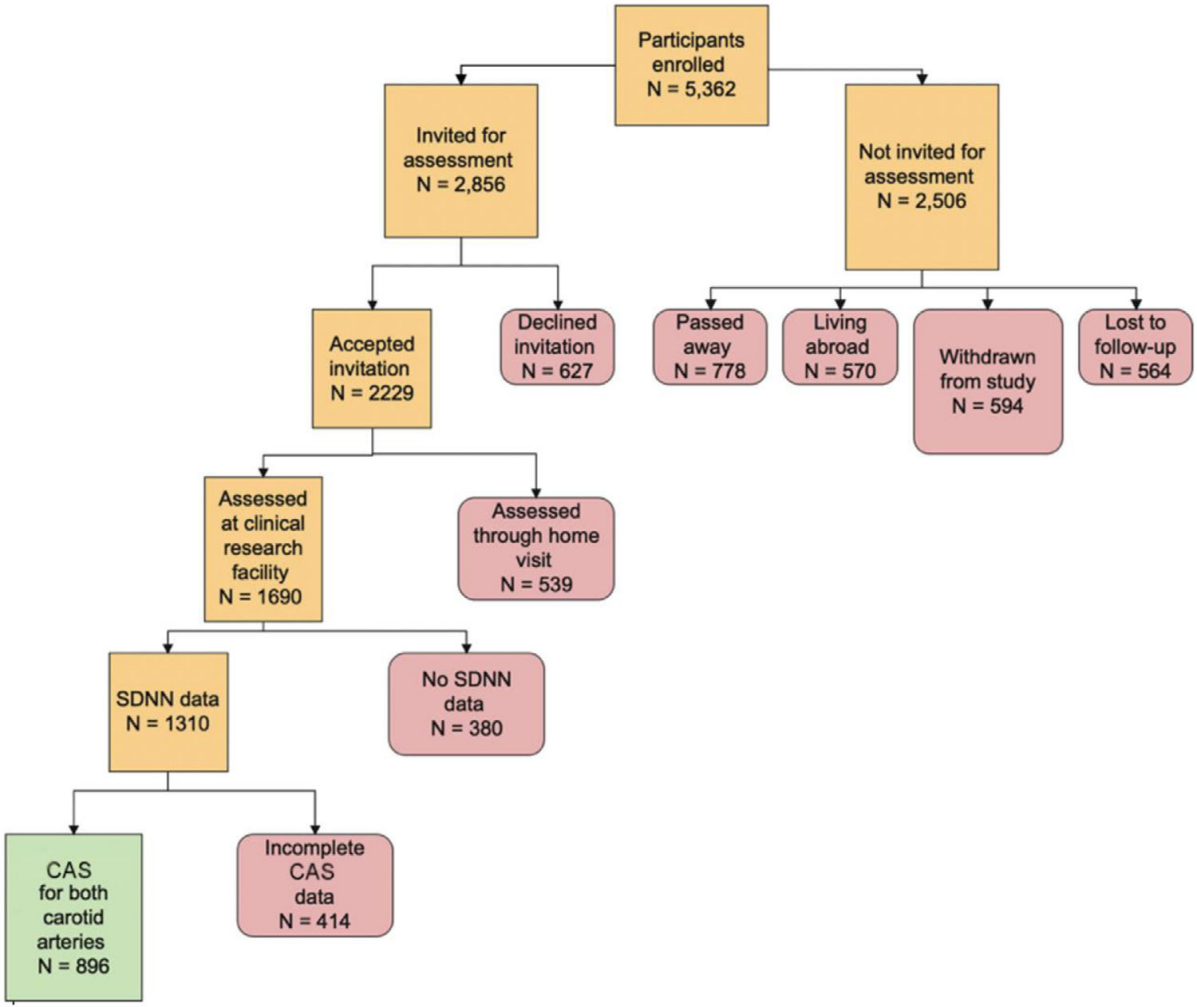
Flowchart summarizing participant inclusion. Loss to follow‐up over time was a concern in NSHD. Those with lower educational attainment, lower childhood cognition, and lifelong smokers were less likely to attend the 60–64 year assessment, but the sample remained representative of the general population as per the 2001 UK census NSHD, National Survey of Health and Development; SDNN, standard deviation of normal‐to‐normal beats.

The participant selection process is shown in Figure 1. The minimum set of inclusion criteria comprised the availability of bilateral CAS ultrasonic data and standard deviation of normal‐to‐normal beats (SDNN) HRV data. Participants were sent a postal invite and pre‐assessment questionnaire. The questionnaire collected data on socio‐demographic factors, lifestyle, and medical history.

### 2.2 Outcome: HRV at 60–64 Years

HRV was assessed through a 5‐min, 12‐lead ECG of the supine, rested participant. The ECG programme was specifically developed for HRV analysis by a member of the data‐gathering team. Recordings were manually cleaned by a physician to remove artifacts, ensure that normal beats were all registered, and that ectopics were discarded. The analysis was completed automatically by CardioNavigator Plus (Spacelabs Healthcare Ltd, Snoqualmie, Washington) to generate values for the following HRV parameters: SDNN, root mean square of successive differences (RMSDD), and high‐frequency (HF) power as measures of parasympathetic activity; low‐frequency (LF) power as a measure of sympathetic activity [10]. Additional indices also included normalized LF, normalized HF power, LF/HF ratio, HRV triangular index, total power spectral density (total PSD), and power spectral density squared.

### 2.3 Exposures

CAS and cIMT were measured at the clinic visit with a GE Vivid I ultrasound scanner (GE Healthcare; Chalfont St Giles, UK) with a high‐resolution probe (12 Hz). Clear images of the carotid artery, 1 cm proximal to the bifurcation, were obtained in three different views (anterior, posterior, and lateral). Ten‐second cineloops were recorded in digital imaging and communications in medicine format and downloaded for offline analysis by the Vascular Physiology Unit, Institute of Cardiovascular Science, University College London, using dedicated software (Carotid Analyser, Iowa City, Iowa).

Images were calibrated, and software was used to automatically generate diameter measurements. Three end‐diastolic frames were selected for each obtained view of the carotid artery. All images were then analyzed by one of two trained and blinded readers. Area strain (given as a percentage) was calculated as the difference between the maximum and minimum cross‐sectional area, as a proportion of the minimum cross‐sectional area. Average strain was calculated as the mean of the left and right carotid artery strain.

cIMT was calculated as previously described [19]. Intra‐ and interobserver reproducibility between readers was evaluated on a subset of 10 randomly selected images. Both inter‐ and intraclass correlations were >0.9, as previously reported [19].

**FIGURE 2:**
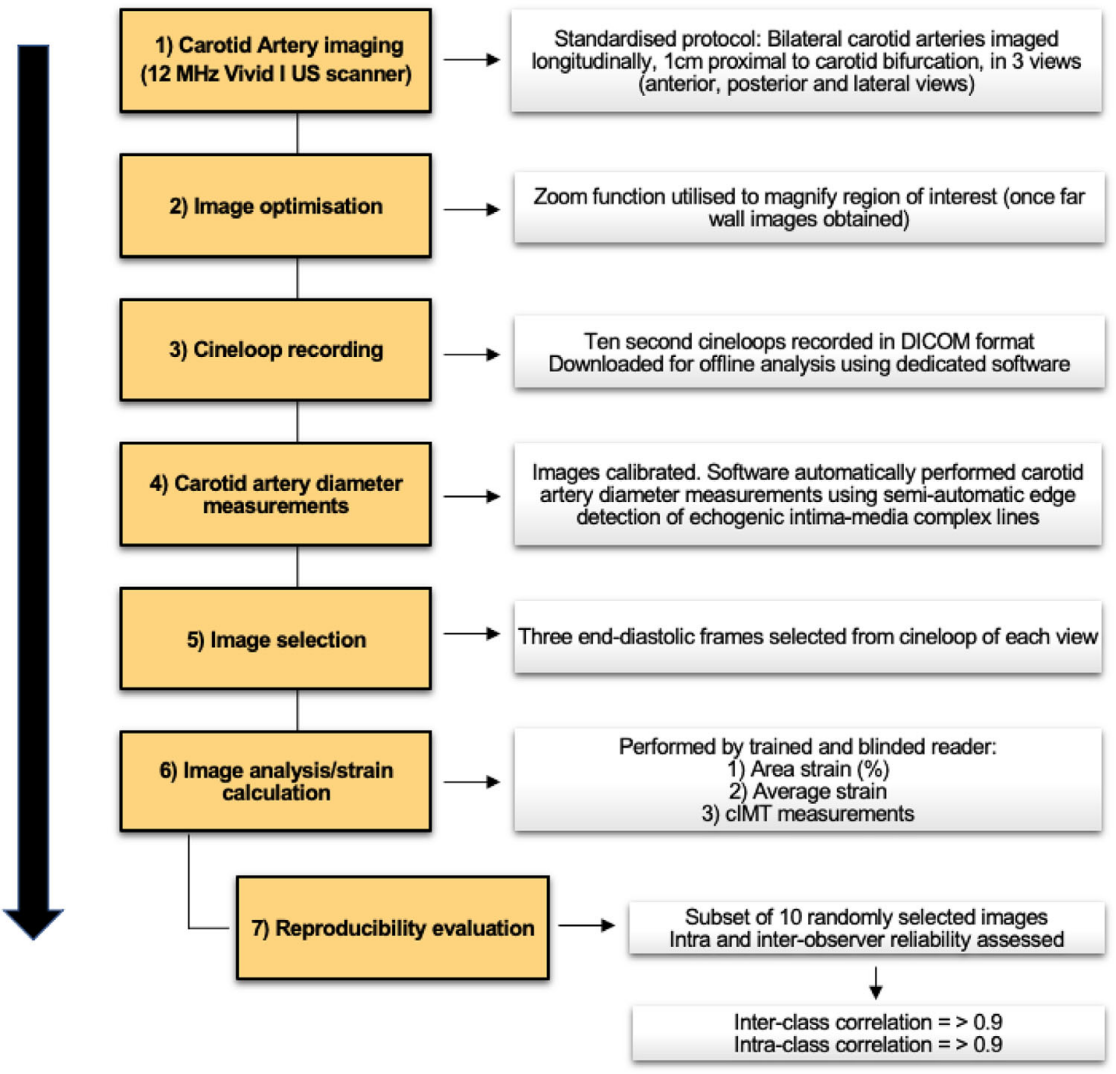
Flowchart summarising step‐by‐step carotid artery imaging and strain analysis methodology. cIMT, carotid intima‐media thickness; DICOM, digital imaging and communication.

CAS/cIMT measurement methodology is further illustrated in Figure 2.

### 2.4 Covariates

Covariates were selected a priori based on their previously published association with HRV and added into our models successively, after centring on age, to help with the interpretation of coefficients. Model 1 adjusted for sex; Model 2 included additional adjustments for SEP; Model 3 added clinical covariates known to be associated with HRV; and Model 4 added cardiac covariates known to be associated with HRV. The same models were used for all the HRV outcomes.

The sex of participants was recorded as male or female (1/2). Height and weight measurements were taken in light, indoor clothing without shoes. Height was measured to the nearest millimetre using a portable stadiometer with the head in the Frankfort plane. Weight measurements to the nearest 0.1 kg were taken to calculate body mass index (BMI). Waist circumference measurements were taken at the midpoint between the costal margin and the iliac crest, and hip circumference was measured at the level of the greater trochanter. The waist‐to‐hip ratio (WHR) was subsequently derived. Participants’ socio‐economic position (SEP) was evaluated using occupational data from 1989, when they were in active working age, according to the UK Office of Population Censuses and Surveys Registrar General's social class, dichotomized as manual or nonmanual (0/1). Brachial systolic and diastolic blood pressure measurements were taken twice with the participant in a seated position using an Omron HEM‐705 sphygmomanometer (OMRON UK Healthcare UK Ltd.; Milton Keynes, UK). The second reading (or the first if the second was missing) was used in our analysis. Two‐dimensional transthoracic echocardiography was performed to measure left ventricular ejection fraction and mass as previously described [20].

Information about medication usage relevant to HRV was collected through survey instruments and self‐reporting, along with other relevant clinical information to capture history of diabetes, heart disease (i.e., ischemic heart disease, myocardial infarction, stroke, heart failure, heart rhythm abnormality, congenital heart disease, rheumatic heart disease, and other cardiovascular diseases), hypertension, and smoking as previously described [21, 22, 23]. Physical activity was assessed using a validated self‐report questionnaire and expressed as the average daily time over the preceding year spent in light‐intensity activity (1.5–2.99 METs), a range corresponding to activities such as slow walking and light domestic work.

For biochemical analysis, a 50‐mL blood sample was collected in the clinic using the Sarstedt system (Sarstedt; Nümbrecht, Germany). Total cholesterol, high‐density lipoprotein (HDL) cholesterol, and triglyceride were measured using a Siemens Dimension Xpand analyzer (Siemens plc Healthcare Sector; Frimley, UK) using the manufacturer's assays. HbA1c was analyzed using a TOSOH G7 analyzer (Tosoh Bioscience Ltd; Redditch, UK). Low‐density lipoprotein (LDL) was calculated by the Friedewald equation. A participant was defined as hypercholesterolaemic if LDL > 4.9 mmol/L based on guidance from the National Institute of Health and Care Excellence [24].

### 2.5 Statistical Analysis

Statistical analyses were conducted using R version 3.6.2 (RStudio Team 2020). Distribution of data was assessed using Q–Q plots, histograms, and the Shapiro–Wilk test. Continuous sample variables are expressed as mean ± 1 standard deviation (SD) or median (interquartile range) as appropriate; categorical sample variables, as counts and percent. Differences between groups were tested using analysis of variance (ANOVA) with post hoc Tukey test or else Kruskal–Wallis with post hoc Nemenyi test for normally and non‐normally distributed continuous data, respectively, or with a Chi‐square test for categorical data.

Due to the skewed distribution of HRV parameters, generalized linear models (GLMs) with a gamma distribution and log link were fitted to examine the associations of CAS and cIMT with HRV. Unless otherwise stated, model coefficients (*β*) in results are expressed as percent change per unit increase in exposure (%Δ per unit), calculated as 100 × [exp(*β*)−1]%. To determine whether the associations of CAS with HRV differed by sex, an interaction term for sex, were tested at the 10% significance level, and no interaction was found to justify stratification by sex. Where more than one measure of CAS was significant at univariate analysis, the average CAS was used in the multivariable model. Model assumptions were verified with regression diagnostics. Multicollinearity between final model variables was excluded by demonstrating variance inflation factors <3. Data missingness was minimal in the study sample (Table S1), so multiple imputation was not required. Strength of evidence for an association was assessed on the basis of the size of the regression coefficients, their confidence interval (CI), and the *p* value. All tests were two‐sided.

We ran sensitivity analyses in which we re‐analyzed the association between CAS and HRV biomarkers after removing participants taking antihypertensives or with known cardiovascular disease, and after additional adjustment for cIMT (Tables S2 and S3).

## 3 Results

### 3.1 Participant Characteristics

Of the 5362 originally enrolled into NSHD, 747 were deceased, 570 had emigrated, 853 had withdrawn, and 530 were not contactable, leaving 2662 who were successfully interviewed between 2006 and 2010. Of these, 896 had contemporaneous 5‐min ECG for SDNN HRV (outcome) and carotid ultrasonography for CAS (exposure, Figure 1). At this data sweep, 170 participants (19%) were on antihypertensives (including beta‐blockers or calcium channel blockers). The remaining characteristics of study participants are presented in Table 1. The population mean for SDNN was 30.0 (IQR 23.1–39.6) with 46.5% being male. Participants with dampened HRV (lowest SDNN quartile) were more likely to be older, male, and smokers, suffering from hypertension, cardiovascular disease, diabetes, or hypercholesterolaemia. Data missingness for key covariates used in multivariable models per exposure–outcome pair is presented in Table S1.

**TABLE 1:** Clinicodemographic characteristics of the cohort according to SDNN quartiles.

Characteristics	All participants *N* = 896	Quartiles of SDNN				*p* value
		Q1, *n* = 224	Q2, *n* = 224	Q3, *n* = 224	Q4, *n* = 224	
**HRV parameters**						
SDNN	30.0 (23.1–39.6)	19.7 (17.1–21.3)	26.5 (24.9–28.3)	34.2 (31.7–36.9)	48.4 (42.9–57.1)	**<0.001**
RMSDD (ms)	18.6 (13.4–26.2)	11.6 (9.2–14.5)	16.8 (13.8–20.3)	21.4 (17.0–26.8)	30.6 (23.6–41.5)	**<0.001**
HRV triangular index	7.8 (6.3–9.8)	5.5 (4.9–6.1)	7.3 (6.6–7.8)	8.9 (7.9–9.5)	11.5 (10.1–13.0)	**<0.001**
Normalized HF power	35.2 (23.0–49.9)	32.8 (21.2–45.9)	34.7 (23.1–48.7)	37.4 (25.2–52.8)	35.6 (24.0–53.3)	0.562
HF power (ms^2^)	109.6 (56.1–218.9)	46.9 (25.1–78.4)	90.0 (56.8–143.9)	140.8 (92.6–275.8)	287.1 (160.4–489.4)	**<0.001**
Power spectral density squared	0.23 (0.18–0.29)	0.22 (0.18–0.28)	0.23 (0.18–0.29)	0.22 (0.17–0.28)	0.24 (0.18–0.31)	0.545
Normalized LF power	64.8 (50.3–77.2)	67.3 (54.2–78.9)	65.6 (51.5–77.1)	62.6 (47.2–75.4)	64.0 (46.8–76.0)	0.569
LF power (ms^2^)	195.7 (109.9–355.7)	90.1 (64.3–121.2)	168.7 (117.9–231.6)	247.6 (167.5–347.0)	477.60 (351.10–723.00)	**<0.001**
LF/HF	1.8 (1.0–3.4)	2.1 (1.2–3.7)	1.9 (1.1–3.3)	1.7 (0.9–3.1)	1.77 (0.88–3.16)	0.565
Total power spectral density (ms^2^)	697.7 (427.2–1196.5)	305.4 (227.1–389.9)	576.6 (498.4–677.0)	898.0 (764.5–1089.0)	1772.0 (1401.0–2404.0)	**<0.001**
**Carotid variables**						
Cross‐sectional CAS left (%)	13.1 (10.8–15.8)	12.7 (10.4–15.2)	13.0 (10.7–15.7)	13.2 (10.6–15.7)	14.0 (11.6–17.0)	**0.001**
Cross‐sectional CAS right (%)	13.8 (11.2–16.6)	13.2 (10.6–16.1)	13.5 (11.1–15.7)	14.1 (11.3–17.0)	14.4 (11.8–17.3)	**0.001**
Average CAS (%)	13.5 (11.3–16.2)	12.7 (10.6–15.7)	13.2 (11.1–15.6)	13.7 (11.2–16.2)	14.4 (12.0–17.2)	**0.011**
Average cIMT (mm)	0.67 (0.60 – 0.76)	0.66 (0.59–0.75)	0.67 (0.60–0.76)	0.69 (0.61–0.77)	0.67 (0.61–0.76)	0.841
cIMT maximum (mm)	0.73 (0.65–0.82)	0.72 (0.63–0.81)	0.73 (0.65–0.82)	0.75 (0.6–0.84)	0.73 (0.65–0.83)	0.848
Demographics						
Age (years)	62.8 (62.0–63.7)	62.9 (62.5–63.6)	62.9 (62.1–63.5)	62.8 (62.2–63.5)	62.6 (61.7–63.4)	***0.008***
Male, *n* (%)	417 (46.5)	101 (11.3)	98 (10.9)	101 (11.3)	117 (13.1)	0.262
SEP at 43 years (manual)						
Manual, *n* (%)	161 (18.0)	48 (5.4)	33 (3.7)	43 (4.8)	37 (4.1)	0.271
Anthropometrics						
BMI (kg/m^2^)	26.5 (24.1–29.6)	26.9 (24.8–30.0)	26.4 (24.1–29.6)	26.4 (23.7–29.5)	26.05 (23.64–28.92)	0.584
Waist‐to‐hip ratio^n^	0.90 ± 0.08	0.91 ± 0.09	0.90 ± 0.08	0.91 ± 0.07	0.90 ± 0.079	0.440
**Cardiac**						
DBP (mmHg)	77.5 (71.0–84.0)	78.0 (71.0–83.8)	78.0 (70.9–82.5)	77.3 (71.0–84.0)	76.50 (70.50–84.50)	0.900
SBP (mmHg)	135.5 (124.0–147.0)	137.0 (125.2–147.5)	135.0 (125.0–147.5)	135.0 (123.9–146.5)	134.80 (123.00–146.50)	0.896
Heart rate (min^−1^)						
LV mass, g	199.5 (162.1–249.0)	203.8 (165.4–248.6)	197.7 (155.5–244.6)	198.70 (164.44–267.67)	197.76 (161.84–241.77)	0.876
LV ejection fraction biplane (%)	65.0 (60.0–69.3)	64.3 (59.6–70.0)	64.0 (59.6–68.7)	65.23 (60.50–69.25)	65.05 (60.32–69.54)	0.952
**Blood markers**						
Total cholesterol (mmol/L)	5.7 (5.03–6.40)	5.6 (4.9–6.3)	5.7 (5.1–6.4)	5.78 (5.10–6.48)	5.67 (5.03–6.43)	0.725
HDL ratio	3.6 (3.1–4.3)	3.6 (3.1–4.3)	3.6 (3.0–4.3)	3.6 (3.0–4.2)	3.7 (3.1–4.3)	0.966
LDL (mmol/L)	3.6 (2.9–4.1)	3.5(2.8–4.0)	3.6 (2.9–4.3)	3.6 (3.0–4.1)	3.6 (2.9–4.2)	0.701
Triglyceride (mmol/L)	1.1 (0.8–1.5)	1.1 (0.7–1.6)	1.1 (0.8–1.6)	1.1 (0.8–1.5)	1.0 (0.7–1.4)	0.717
HbA1c (mmol/L)	39.0 (37.0–41.0)	40.0 (38.0–42.0)	39.0 (37.0–41.0)	38.5 (36.0–41.0)	39.0 (36.0–41.0)	**0.003**
**Other clinical factors**						
Smoking						
Ex‐smoker, *n* (%)	314 (35.0)	77 (8.6)	84 (9.4)	85 (9.49)	68 (7.59)	0.305
Current smoker, *n* (%)	65 (7.3)	15 (1.7)	14 (1.6)	18 (2.01)	18 (2.01)	0.838
Exercise levels (min/day)	190.9 (120.1–284.6)	198.4 (116.0–296.2)	188.6 (120.4–274.4)	201.4 (117.9–287.1)	180.82 (122.68–281.25)	0.920
MI or angina, *n* (%)	44 (4.9)	17 (1.9)	12 (1.3)	10 (1.1)	5 (0.56)	0.070
Stroke, *n* (%)	8 (0.9)	2 (0.2)	4 (0.5)	0 (0.0)	2 (0.22)	0.440
Diabetes mellitus, *n* (%)	38 (4.2)	13 (1.5)	9 (1.0)	8 (0.9)	8 (0.89)	0.600
Hypertension, *n* (%)	364 (40.6)	104 (11.6)	91 (10.2)	86 (9.6)	83 (9.26)	0.189
Hypercholesterolaemia, *n* (%)	74 (8.3)	11 (1.2)	23 (2.6)	23 (2.6)	17 (1.90)	0.120
History of cardiovascular event, *n* (%)	50 (5.6)	18 (2.0)	15 (1.7)	10 (1.1)	7 (0.78)	0.103

*Note*: Results are reported as counts (%) for categorical variables, mean ± 1 standard deviation for normally distributed variables (^n^) or median (interquartile range) for non–normal variables.

Abbreviations: BMI, body mass index; cIMT, carotid intima‐media thickness; DBP, diastolic blood pressure; HDL, high‐density lipoprotein; HF, high‐frequency; HRV, heart rate variability; LDL, low‐density lipoprotein; LF, low‐frequency; LV, left ventricular; MI, myocardial infarction; Q1/2/3/4, Quartile 1/2/3/4; RMSDD, root mean square of successive differences; SBP, systolic blood pressure; SDNN, standard deviation of normal‐to‐normal beats; SEP, socioeconomic position and occupation type.

Significant *p* values are highlighted in bold (*p* < 0.05).

### 3.2 Associations of CAS With SDNN, RMSDD, and HRV Triangular Index

Left (%Δ per unit = 61.6%, 95% CI [35.0%–101.0% per unit], *p* < 0.001), right (%Δ per unit = 46.3% [22.1%–82.2%], *p* < 0.001), and average (%Δ per unit = 69.8% [35.0%–122.5%], *p* < 0.001) cross‐sectional CAS showed significant positive associations with SDNN on univariate analysis (Table 2), while age (%Δ per unit = −79.0% [–90.0% to –55.1%], *p *< 0.001), sex (%Δ per unit = –84.0% [–97.3% to 0.0%], *p* = 0.047), BMI (%Δ per unit = –20.6% [–33.0% to 0.0%], *p* = 0.025), triglyceride levels (%Δ per unit = –76.3% [–90.0% to –33.0%], *p* = 0.008), HbA1c (%Δ per unit = −18.1% [−25.9% to −9.5%], *p *< 0.001), and previous MI or angina (%Δ per unit = −98.3% [−99.9% to −9.5%], *p* = 0.029) showed significant negative associations with SDNN. In fully adjusted multivariable models, average CAS (%Δ per unit = 68.2% [22.1%–122.6%], *p*<0.001), age (%Δ per unit = −67.1% [−86.5% to −18.1%], *p* = 0.016), and male sex (%Δ per unit = −93.6% [−99.3% to −45.1%], *p* = 0.011) retained independent associations with SDNN (Table 3).

**TABLE 2:** Univariate regression analysis for each exposure or covariate with HRV indices.

	SDNN		RMSDD		HRV triangular index		HF power		LF power		Normalized HF power	
Variable	*β* Coefficient (95% CI)	*p* value	*β* coefficient (95% CI)	*p* value	*β* coefficient (95% CI)	*p* value	*β* coefficient (95% CI)	*p* value	*β* coefficient (95% CI)	*p* value	*β* coefficient (95% CI)	*p* value
**Carotid variables**												
Cross‐sectional CAS left	0.48 (0.3, 0.7)	**<0.001**	0.45 (0.2, 0.7)	**<0.001**	0.10 (0.1, 0.1)	**<0.001**	5.64 (0.7, 11.1)	**0.030**	9.99 (4.4, 16.2)	**0.002**	0.26 (−0.06, 0.58)	0.104
Cross‐sectional CAS right	0.38 (0.2, 0.6)	**<0.001**	0.40 (0.2, 0.6)	**<0.001**	0.08 (0.0, 0.1)	**<0.001**	5.16 (0.6, 9.8)	**0.031**	4.84 (0.2, 10.0)	0.110	0.30 (0.00, 0.61)	**0.046**
Average CAS	0.53 (0.3, 0.8)	**<0.001**	0.53 (0.3, 0.8)	**<0.001**	0.11 (0.1, 0.2)	**<0.001**	6.97 (1.5, 12.8)	**0.012**	8.50 (2.8, 14.8)	**0.014**	0.35 (0.01, 0.71)	**0.040**
Average cIMT	2.7 (−4.7, 10.3)	0.474	3.44 (−3.5, 10.7)	0.327	0.10 (−1.4, 1.6)	0.898	39.2 (−119.8, 214.5)	0.627	17.12 (−178.6, 227.4)	0.869	0.76 (−9.05, 11.04)	0.880
cIMT maximum	2.39 (−3.6, 8.6)	0.439	2.83 (−2.8, 8.7)	0.324	0.09 (−1.1, 1.3)	0.884	47.00 (−83.7, 190.4)	0.483	10.66 (−149.2, 185.2)	0.900	0.37 (−7.63, 8.80)	0.929
**Demographics**												
Age	−1.56 (−2.3, −0.8)	**<0.001**	−0.37 (−1.1, 0.3)	0.314	−0.37 (−0.5, −0.2)	**<0.001**	−10.80 (−29.2, 5.3)	0.228	−23.13 (‐44.7, −3.6)	**0.029**	0.48 (−0.58,1.50)	0.357
Male	−1.83 (−3.6, 0.0)	**0.047**	0.57 (−1.1, 2.2)	0.499	−0.18 (−0.6, 0.2)	0.348	34.56 (−3.2, 72.7)	0.071	−47.51 (−98.6, 1.4)	0.061	6.79 (4.42,9.18)	**<0.001**
SEP at 43 years (manual)	0.24 (−2.0, 2.7)	0.841	0.34 (−1.8, 2.7)	0.761	−0.21 (−0.7, 0.3)	0.391	2.12 (−43.8, 59.0)	0.934	−34.92 (−92.8, 33.3)	0.270	2.70 (−0.51,6.16)	0.111
**Anthropometrics**												
BMI	−0.23 (−0.4, 0.0)	**0.025**	−0.02 (−0.2, 0.2)	0.803	−0.04 (−0.1, 0.0)	**0.039**	2.01 (−2.2, 6.5)	0.377	−4.09 (−8.7, 1.3)	0.133	0.27 (0.01, 0.55)	0.059
Waist‐to‐hip ratio	−4.70 (−16.0, 6.7)	0.420	−6.92 (−17.4, 3.5)	0.194	−1.54 (−3.9, 0.8)	0.194	−190.30 (−428.3, 47.7)	0.117	−131.50 (−443.0, 179.9)	0.407	−16.56 (−31.06, −1.91)	**0.032**
**Cardiac**												
LV mass	0.00 (0.0, 0.0)	0.964	0.01 (0.0, 0.0)	0.335	0.00 (0.0, 0.0)	0.225	−0.96 (−2.7, 0.9)	0.328	0.42 (−2.1, 3.0)	0.747	−0.18 (−0.30, −0.06)	**0.004**
LV ejection fraction biplane	−0.04 (−0.2, 0.1)	0.598	−0.01 (−0.1, 0.1)	0.892	0.0 (0.0, 0.0)	0.847	−0.39 (−1.3, 0.7)	0.454	0.30 (−1.0, 1.6)	0.659	−0.06 (−0.12, 0.00)	0.056
Mean DBP	−0.03 (−0.1, 0.1)	0.578	−0.08 (−0.2, 0.0)	0.074	−0.01 (0.0, 0.0)	0.392	0.09 (−0.2, 0.4)	0.598	0.19 (−0.2, 0.6)	0.321	0.00 (−0.02, 0.02)	0.727
Mean SBP	−0.01 (−0.1, 0.0)	0.661	−0.03 (−0.1, 0.0)	0.200	0.00 (0.0, 0.0)	0.575	0.64 (−2.2, 3.2)	0.641	−0.22 (−3.6, 2.9)	0.906	0.11 (−0.07, 0.29)	0.216
**Blood markers**												
Total cholesterol	0.16 (0.0, 1.0)	0.695	−0.26 (−1.0, 0.5)	0.497	0.06 (−0.1, 0.2)	0.507	−6.96 (−21.8, 8.9)	0.410	6.71 (−16.7, 30.5)	0.558	−0.33 (−1.35, 0.71)	0.544
HDL ratio	0.06 (−1.0, 1.1)	0.900	−0.39 (−1.3, 0.5)	0.400	0.00 (−0.2, 0.2)	0.979	−11.47 (−27.1, 9.3)	0.248	−5.99 (−33.0, 23.9)	0.663	−0.64 (−1.87, 0.67)	0.344
LDL	0.56 (−0.4, 1.5)	0.249	−0.0 (−0.9, 0.8)	0.962	0.13 (−0.1, 0.3)	0.184	−4.10 (−21.9, 14.8)	0.683	11.89 (−16.5, 40.6)	0.383	−0.35 (−1.55, 0.87)	0.585
Triglyceride	−1.44 (−2.3, −0.4)	**0.008**	−1.12 (−1.8, −0.2)	**0.019**	−0.30 (−0.5, −0.1)	**0.009**	−15.52 (−34.6, −5.6)	**0.002**	−30.76 (−59.0, −1.6)	**0.039**	−0.75 (−2.16, 0.90)	0.338
HbA1c	−0.20 (−0.3, −0.1)	**<0.001**	−0.10 (−0.2, 0.0)	0.060	−0.05 (−0.1, 0.0)	**<0.001**	−1.40 (−2.5, 1.7)	0.196	−1.61 (−2.1, −1.2)	**<0.001**	0.09 (−0.10, 0.31)	0.358
**Other clinical factors**												
Smoking	−0.47 (−1.9, 1.0)	0.531	−0.61 (−2.0, 0.7)	0.386	0.07 (−0.2, 0.4)	0.640	−16.27 (−48.4, 12.2)	0.329	−20.09 (−67.8, 17.6)	0.357	−0.91 (−2.92, 1.03)	0.373
Exercise levels	0.00 (0.0, 0.0)	0.736	0.00 (0.0, 0.0)	0.624	0.00 (0.0, 0.0)	0.686	0.08 (−0.1, 0.2)	0.324	−0.08 (−0.3, 0.1)	0.412	0.01 (0.01, 0.02)	**0.003**
MI or angina	−4.10 (−7.5, −0.1)	**0.029**	1.76 (−2.1, 6.6)	0.422	−0.76 (−1.5, 0.1)	0.066	42.93 (−41.1, 191.6)	0.436	−78.84 (−156.7, 43.0)	0.102	3.82 (−1.87, 10.65)	0.229
Stroke	−2.71 (−10.0, 8.0)	0.543	−1.07 (−1.6, −0.1)	**0.002**	−0.27 (−0.4, 0.0)	**0.009**	−16.07 (−23.4, −8.7)	**<0.001**	−22.84 (−35.2, −10.5)	**<0.001**	−0.47 (−1.71, 1.77)	0.552
Diabetes mellitus	−2.91 (−6.7, 1.5)	0.163	−0.75 (−4.3, 3.7)	0.712	−0.56 (−1.4, 0.4)	0.200	11.88 (−66.4, 159.3)	0.821	−106.20 (−175.6, 6.5)	**0.015**	1.55 (−4.36, 8.81)	0.642
Hypertension	−1.46 (−3.3, 0.4)	0.115	−1.67 (−3.3, 0.0)	0.051	−0.31 (−0.7, 0.1)	0.101	−22.43 (−60.0, 16.2)	0.243	−6.34 (−56.1, 45.5)	0.805	−2.35 (−4.78, 0.11)	0.059
Hypercholesterolaemia	1.29 (−1.9, 4.9)	0.454	1.19 (−1.7, 4.6)	0.457	0.31 (−0.4, 1.0)	0.377	34.68 (−30.3, 132.0)	0.383	−23.15 (−94.7, 75.3)	0.582	2.37 (−1.91, 7.24)	0.309
History of cardiovascular event	−3.48 (−6.8, 0.3)	0.054	1.42 (−2.1, 5.7)	0.465	−0.54 (−1.3, 0.3)	0.164	60.49 (−24.1, 205.5)	0.270	−62.80 (−138.0, 52.6)	0.175	4.41 (−1.15, 11.05)	0.154

*Note*: Significant *p* values are highlighted in bold (*p* < 0.05).

Abbreviations: BMI, body mass index; cIMT, carotid intima‐media thickness; DBP, diastolic blood pressure; HDL, high‐density lipoprotein; HF, high‐frequency; HRV, heart rate variability; LDL, low‐density lipoprotein; LF, low‐frequency; LV, left ventricular; MI, myocardial infarction; Q1/2/3/4, Quartile 1/2/3/4; RMSDD, root mean square of successive differences; SBP, systolic blood pressure; SDNN, standard deviation of normal‐to‐normal beats; SEP, socioeconomic position and occupation type.

**TABLE 3:** Multivariable regression analysis for CAS with HRV indices.

	SDNN		RMSDD		HRV triangular index		HF power		LF power		Normalised HF power	
Variable	*β* coefficient (95% CI)	*p* value	*β* coefficient (95% CI)	*p* value	*β* coefficient (95% CI)	*p* value	*β* coefficient (95% CI)	*p* value	*β* coefficient (95% CI)	*p* value	*β* coefficient (95% CI)	*p* value
Age	−1.11 (−2.0, −0.2)	**0.016**	−0.12 (−1.0, 0.7)	0.781	−0.34 (−0.5, −0.1)	**<0.001**	−6.23 (−23.8, 11.3)	0.486	−8.17 (−31.8, 15.4)	0.497	0.39 (−0.8, 1.5)	0.507
Sex	−2.75 (−4.9, −0.6)	**0.011**	0.44 (−1.6, 2.5)	0.670	−0.34 (−0.8, 0.1)	0.149	34.98 (−7.6, 77.5)	0.107	−62.80 (−117.6, −7.9)	**0.025**	7.61 (4.8, 10.4)	**<0.001**
Average CAS	0.52 (0.2, 0.8)	**0.001**	0.59 (0.3, 0.9)	**<0.001**	0.09 (0.0, 0.2)	**0.005**	8.33 (2.2, 14.4)	**0.007**	8.47 (1.0, 15.9)	**0.026**	0.55 (0.2, 0.9)	**0.006**
SEP at 43 years	−0.10 (−2.7, 2.6)	0.942	−1.59 (−3.9, 1.0)	0.198	−0.26 (−0.8, 0.3)	0.3705	−28.50 (−74.9, 17.9)	0.228	−18.43 (−80.7, 43.9)	0.562	−0.46 (−3.9, 3.3)	0.800
BMI	−0.06 (−0.3, 0.2)	0.646	0.05 (−0.2, 0.3)	0.730	−0.02 (−0.1, 0.0)	0.486	2.56 (−3.1, 8.2)	0.375	−4.06 (−10.2, 2.1)	0.194	0.11 (−0.3, 0.5)	0.577
Triglyceride	−0.94 (−2.4, 0.6)	0.207	−1.10 (−2.4, 0.4)	0.109	−0.19 (−0.5, 0.2)	0.252	−19.69 (−43.1, 3.7)	0.098	−6.61 (−38.3, 25.0)	0.682	−0.72 (−2.6, 1.4)	0.466
HbA1c	−0.10 (−0.2, 0.0)	0.143	−0.03 (−0.1, 0.1)	0.634	−0.03 (−0.1, 0.0)	0.061	−0.18 (−2.9, 2.6)	0.900	−1.06 (−3.3, 1.2)	0.350	0.08 (−0.1, 0.3)	0.461
MI/angina	−2.46 (−6.8, 2.7)	0.302	2.81 (−2.0, 9.0)	0.298	−0.37 (−1.4, 0.8)	0.485	107.56 (−52.2, 267.4)	0.187	26.62 (−78.0, 131.3)	0.618	5.92 (−1.1, 14.6)	0.134
Stroke	−0.89 (−1.8, 0.7)	0.142	−0.68 (−1.5, 1.2)	0.230	−0.14 (−0.4, 0.2)	0.339	−9.18 (−26.6, 8.2)	0.300	−15.55 (−35.8, 4.7)	0.132	−0.62 (−2.0, 2.1)	0.524
Hypertension	−1.59 (−3.8, 0.6)	0.157	−1.33 (−3.4, 0.8)	0.217	−0.34 (−0.8, 0.2)	0.175	−13.43 (−57.2, 30.3)	0.546	−36.02 (−91.1, 19.1)	0.200	−1.43 (−4.4, 1.6)	0.341

*Note*: Only fully adjusted data for the final model (Model 4) are shown. Model 1 (centered on age) adjusted for sex; Model 2 additionally adjusted for SEP; Model 3 additionally adjusted for clinical covariates, namely BMI, HBA1c, and triglycerides; Model 4 additionally adjusted for cardiac covariates, namely, hypertension, prior stroke, and previous MI or angina.

Abbreviations: BMI, body mass index; cIMT, carotid intima‐media thickness; DBP, diastolic blood pressure; HDL, high‐density lipoprotein; HF, high‐frequency; HRV, heart rate variability; LDL, low‐density lipoprotein; LF, low‐frequency; LV, left ventricular; MI, myocardial infarction; Q1/2/3/4, Quartile 1/2/3/4; RMSDD, root mean square of successive differences; SBP, systolic blood pressure; SDNN, standard deviation of normal‐to‐normal beats; SEP, socioeconomic position and occupation type.

Significant *p* values are highlighted in bold (*p *< 0.05).

On univariate analysis, average, left, and right CAS, respectively, were all positively associated with RMSDD (%Δ per unit = 69.8% [35.0%–122.6%], *p *< 0.001; = 56.8% [22.1%–101.4%], *p *< 0.001; = 49.2% [22.1%–82.2%], *p *< 0.001). Triglyceride and stroke, respectively, were negatively associated with RMSDD (%Δ per unit = −67.4% [−83.5% to −18.1%], *p* = 0.019; = −65.7% [−79.8% to −9.5%], *p* = 0.002) (Table 2). In fully adjusted multivariable models, only average CAS retained an independent association with RMSDD (%Δ per unit = 80.4% [35.0%–146.0%], *p *< 0.001, Table 3).

On univariate analysis, average, left, and right CAS, respectively, were all positively associated with HRV triangular index (%Δ per unit = 11.6% [10.5%–22.1%], *p* < 0.001; = 10.5% [10.5%–10.5%], *p *< 0.001; = 8.3% [0.0%–10.5%], *p *≤ 0.001, Table 2). Age (%Δ per unit = −30.9% [−39.4% to −18.1%], *p *< 0.001), BMI (%Δ per unit = −25.9% [−39.4% to −9.5%], *p* = 0.039), triglyceride (%Δ per unit = −25.9% [−39.4% to −9.5%], *p* = 0.009), HbA1c (%Δ per unit = −4.9% [−9.5% to 0.0%], *p *< 0.001), and stroke (%Δ per unit = −23.7% [−33.0% to 0.0%], *p* = 0.009) were negatively associated with HRV triangular index. In fully adjusted multivariable models, only age and average CAS retained independent associations (%Δ per unit = −28.8% [−39.4% to −9.5%], *p *< 0.001; = 9.42% [0.0%–22.1%], *p* = 0.005, Table 3).

### 3.3 Associations of CAS With HF Power, LF Power, and Normalized HF Power

Average, left, and right CAS showed a significant positive association with HF power at univariate analysis (respectively %Δ per unit = 10.6 × 10^4^% [3.5 × 10^2^%–36.2 × 10^6^%], *p* = 0.012; = 28.0 × 10^3^% [101%–66.2 × 10^5^%], *p* = 0.031; = 17.3 × 10^3^% [82.2%–180.3 × 10^4^%], *p* = 0.030, Table 2). On multivariable analysis, average CAS (%Δ per unit = 41.5 × 10^4^% [802%–17.9 × 10^7^%], *p* = 0.007) retained a significant association (Table 3).

Average (%Δ per unit = 49.2 × 10^4^% [15.4 × 10^2^%–26.8 × 10^7^%], *p *< 0.014) and left (%Δ per unit = 21.8 × 10^5^% [8.0 × 10^3^%,10.8 × 10^8^%], *p* = 0.002) CAS were significantly associated with LF power on univariate analysis. HbA1c was negatively associated with LF power (%Δ per unit = −80% [−87.8% to −69.9%], *p *< 0.001), as were age, triglyceride blood levels, previous stroke, and diagnosis of diabetes mellitus (Table 2). On multivariable analysis, average CAS (%Δ per unit = 47.7% [17.1 × 10^1^%, 80.4 × 10^7^%], *p* = 0.026) was significantly associated with LF power (Table 3).

Average and right CAS were associated with normalized HF power at univariate analysis (%Δ per unit = 41.9% [1.0%–103.4%], *p* = 0.040; = 35.0% [0.0%–84.0%], *p* = 0.046). Other associations are summarized in Table 2. Average CAS and sex retained significance in fully adjusted multivariable models (%Δ per unit = 73.3% [22.1%–146.0%], *p* = 0.006; = 20.2 × 10^4^% [12.1 × 10^3^%–32.9 × 10^5^%], *p *< 0.001; Table 3).

The association between average CAS with total power spectral density and PSD squared was significant at univariate analysis but attenuated after multivariable adjustment (Tables S4 and S5). There was no association between normalized LF power and LF/HF ratio with average CAS at univariate analysis.

### 3.4 Sensitivity Analysis

When removing participants with known cardiovascular disease and antihypertensives from the analysis, average CAS retained association with SDNN, RMSDD, and HRV triangular index (%Δ per unit = 53.7% [10.5%–101.4%], *p* = 0.003; = 63.2% [22.1%–122.6%], *p* < 0.001; = 7.25% [0.0%–10.5%], *p* = 0.021, Table S2) and the same was observed after adjusting for cIMT (Table S3).

## 4 Discussion

In a cross‐sectional population‐based study, we found that older persons with stiffer carotids exhibited impairment of normal HRV.

Our study data show an independent association between CAS and several HRV measures, including SDNN, RMSDD, HRV triangular index, HF/LF power, and normalized HF power. Results lend credence to our initial theory that reduced CAS could potentially dampen the sensitivity of carotid sinus baroreceptors, thus reducing HRV. This is also consistent with previous studies reporting similar associations in younger cohorts [16, 17], healthy adults [25], in patients with hypertension [26], and in patients with type 2 diabetes mellitus [27].

Carotid vascular stiffness indices are significantly associated with endothelial dysfunction as measured by flow‐mediated dilatation [28]. Given that endothelial changes precede atherosclerosis and correlate with disease severity in both early and late stages, carotid CAS may be a more sensitive atherosclerosis biomarker than cIMT. This may explain the evidence base confirming the incremental benefit of CAS measurement in atherosclerotic risk prediction models compared to cIMT measurement alone [4, 5, 6, 7]. There is little evidence to support the role of lipid‐lowering interventions on CAS; however, a ketogenic diet that increases LDL has been shown to associate with a decrease in carotid distensibility (though not in cIMT) in patients with difficult‐to‐treat epilepsy [29]. Therefore, CAS may better reflect short‐term changes in atherosclerotic risk factors compared to cIMT.

The majority of HRV parameters we appraised in this study showed association with both left and right CAS, with the sole exception of normalized HF power. The asymmetrical cardiac reflex response has long been recognized, but data are conflicting with some studies that focused on the RR interval, reporting greater dependence on right carotid sinus stimulation [30, 31], while another study found no left‐right differences in the carotid–cardiac reflex responses [32]. The fairly consistent asymmetry identified in our study could be related to differences in right/left‐sided cardiac innervation and to different projections of baroreceptor afferents to the solitary tract nucleus [31]. Because stimulation of the right carotid sinus in various clinical scenarios may have a larger influence on RR interval variability, this may confound the association with HRV biomarkers for a given value of right CAS compared to the left, resulting in a stronger association for the left than the right, as seen in our study.

We found that although LF and HF power were associated with CAS, the LF/HF ratio was not. This is in agreement with another study assessing HRV and carotid vascular stiffness indices in hypertensive patients [26]. While LF and HF power increase as CAS increases, the rate of increase is such that the ratio between the two remains unchanged. LF and HF power were previously thought to reflect sympathetic and parasympathetic tone, respectively [33], but this view has been fairly strongly criticized. Our results would suggest that the reduced CAS affects both systems equally, so sympathovagal balance is maintained. This is contentious, however, and it is likely that there is no such well‐defined boundary between the representation of sympathetic and parasympathetic tone in HRV analysis. Recent studies suggest that LF power may be reflecting cardiac autonomic outflow by baroreflexes rather than true sympathetic tone [34, 35], in which case LF and HF power may be capturing nondistinct determinants of HRV, detracting from our ability to infer the sympathovagal balance.

We found a significant association between CAS and HRV but not between cIMT and HRV, despite both CAS and cIMT being putative biomarkers of carotid atherosclerotic severity [4, 7]. The published literature is similarly divided, with some previous studies describing a significant inverse relationship between cIMT and HRV [36, 37] and others finding no significant association [38, 39]. Discrepant results could be attributed to study design differences, with some factors such as participants’ age, physical activity levels, and other clinically relevant covariates not being adjusted for where associations were reported. The baroreflex is initiated in response to baroreceptor stretch in the carotid sinus. cIMT does not closely relate to arterial stiffness until an advanced pathological degree of thickening is reached [40, 41]. Early cIMT thickening may not alter the stretch of the baroreceptor, thus weakening the observed association with HRV, explaining our study findings.

### 4.1 Confounding Variables

Type 2 diabetes: Previous studies only identified an association between carotid vascular stiffness indices and total power, LF or HF power in patients with type 2 diabetes when adjustment for cardiac autonomic neuropathy (CAN) was performed [27, 42]. Our study found that HbA1c—a summary measure of blood glucose levels over the preceding 3 months—was significantly associated with SDNN, HRV triangular index, and LF power at univariate analysis and tended towards significance along with HRV triangular index at multivariable analysis, suggesting a potentially significant biological association between HbA1c and HRV.

Previous stroke: Our study did not find an association between previous stroke and HRV in contrast to other studies [43, 44], likely due to the small stroke numbers in our cohort.

Triglycerides/Hypercholesterolaemia: Our results demonstrated a significant association between HRV and plasma triglyceride levels at univariate analysis, but not with LDL or HDL, replicating findings from another study on nondiabetic individuals [45].

Physical activity: We did not find a significant association between physical activity levels and HRV, in contrast to a recent meta‐analysis [46]. This discrepancy could be due to the age of our cohort, with generally low levels of physical activity being reported. It could also be explained by the subjective self‐reported physical activity measures used in our study, compared to more objective approaches used by others.

### 4.2 Limitations

Given the cross‐sectional study design, it is not possible to imply causality from the observed associations. The inclusion of British people born during the same week in 1946 leads to issues with external validity as the data cannot be easily generalized to non‐British populations. Arterial stiffness is highly dependent on blood pressure, and we did not pursue recently derived formulae to calculate an arterial stiffness index independent of blood pressure [47]. The addition of carotid plaque status to the current analysis would have provided important mediating or confounding insights into observed associations, but these data are not currently available in NHSD and will be explored as part of future work. Additionally, the absence of long‐term ECG HRV data for this cohort restricted our ability to comment upon any association between CAS and Ultra‐Low Frequency HRV data and reduced the overall reliability of our frequency domain data [10, 11].

We acknowledge substantial heterogeneity of self‐reported medication use in our cohort, including in prescribed dosages, unknown underlying indications for prescription of medications, and uncertain adherence to reported medications. For this reason, and because of multicollinearity with hypertension and cardiovascular disease covariates, medications were not included in multivariable models, and we showed in sensitivity analyses (Table S2) that excluding subjects on beta‐blockers and calcium channel blockers did not significantly alter the observed effect estimates.

## 5 Conclusion

Regardless of the presence of carotid atherosclerotic vascular disease (indicated by cIMT), hypertension or stroke, carotid stiffening in older age associates with a dampened HRV response, potentially through an impaired baroreceptor response.

## Author Contributions

All authors contributed significantly to the design, implementation, analysis, interpretation, and manuscript writing. The corresponding author attests that all listed authors meet the authorship criteria and that no others meeting the criteria have been omitted.

## Funding

G.C. has received support in the form of a special project grant from the British Heart Foundation with reference SP/20/2/34841 and by the NIHR UCL Hospitals Biomedical Research Centre. The NSHD cohort is funded by the UK MRC (program codes MC_UU_12019/1; MC_UU_12019/4; MC_UU_12019/5). J.C.M. is directly and indirectly supported by the UCL Hospitals NIHR BRC and Biomedical Research Unit at Barts Hospital, respectively. A.D.H. receives support from the British Heart Foundation, the Economic and Social Research Council (ESRC), the Horizon 2020 Framework Programme of the European Union, the National Institute on Aging, the National Institute for Health Research, University College London Hospitals Biomedical Research Centre, and the UK MRC.

## Conflicts of Interest

The authors declare no conflicts of interest.

## Supporting information




**Supporting File 1**: jch70312‐sup‐0001‐SupMat.docx

## Data Availability

The data that support the findings of this study are available in the supporting material of this article. NSHD data are available from https://www.nshd.mrc.ac.uk/data. Data spreadsheets and statistical codes used for this analysis are provided online in GitHub https://github.com/MaxFornasiero/HRVxCarotidDistensibility/blob/main/Main.
